# Temperature and settlement characteristics of graded crushed-rock layer for runway engineering in permafrost regions

**DOI:** 10.1371/journal.pone.0274843

**Published:** 2022-10-26

**Authors:** Xiaolan Liu, Chuanwei Fu, Shunqun Li

**Affiliations:** Tianjin Key Laboratory of Civil Structure Protection and Reinforcement, Tianjin Chengjian University, Tianjin, China; Al Mansour University College-Baghdad-Iraq, IRAQ

## Abstract

To maintain the temperature and settlement stability of a subgrade, a combination of graded crushed rock layer and insulation layer may be applied to optimize pavement structures. This study verifies a proposed numerical model of pavement and subgrade design in permafrost regions and evaluates temperature and settlement characteristics at different particle size and thickness of graded crushed rock layer and different thickness and location of insulation layer. The results show that the temperature and settlement of the combination of graded crushed-rock layer and insulation layer decrease significantly as the particle diameter and thickness of graded crushed-rock layer increase, and vary little when the thickness of insulation layer is more than 0.15 m. The location of installation layer has significant influence on the temperature of the subbase layer, but has little influence on the temperature of the subgrade. The maximum settlement of the pavement structure and subgrade decreases when the installation layer varies from the top of the subbase layer to the bottom of the subbase layer. The optimal combination of graded crushed-rock layer and insulation layer is that the 0.15 m-thickness installation layer is at the bottom of the 0.50 m-thickness graded crushed-rock layer with the particle size of 6–8 cm. This study provides a theoretical basis for the design, construction, operation, maintenance, and safety management of airport runways in permafrost regions.

## 1. Introduction

The thermal stability of permafrost is poor, and its settlement frequently causes the cracking, deformation, subsidence, and collapse of engineered structures [1, 2]. Hence, there is an urgent need to improve the thermal stability of runways in permafrost regions. Existing optimization techniques for maintaining subgrade structure stability include the use of rock, thermal probe, ventilation pipeline, crushed-rock, and insulation subgrade [3]. Rock subgrade is laid on the bedrock directly, and its stability can only be controlled by selecting a location with minimal to no permafrost [3]. Hence, rock subgrades have high geological and topographical requirements; it is difficult to ensure the safety of aircraft landing and takeoff, making this measure suitable for small airports with short runways only. A thermal probe subgrade is a tubular cooling device that uses liquid-gas two-phase medium convection circulation to dissipate heat [4, 5]. However, this subgrade is only suitable for a limited time when the outside air temperature is lower than that of the subgrade. Moreover, the insertion length and angle, external radiator characteristics, arrangement scheme, and elevation of the exposed part of the thermal probe can impact aircraft safety. The ventilation pipeline subgrade exchanges heat by convection and conduction and then influences the temperature field by the heat conduction effect of the pipe wall material to control permafrost subgrade stability [6, 7]. However, the construction of the ventilation pipeline subgrade is complex with high maintenance costs, and such subgrade is difficult to maintain and replace. Because both the crushed-rock subgrade and insulation subgrade are convenient and cheap to construct with minimal effect on aircraft safety, an increasing number of researchers have studied the application of crushed-rock and insulation subgrade in airport runways.

The crushed-rock subgrade method uses crushed-rock to replace shallow permafrost, which promotes subgrade stability. For example, 1.5 m-thick crushed-rock subgrade was used in the Alaska Arctic Coastal Plain Airport, 1.8 m-thick crushed-rock subgrade was used in the Brookes Mountain Area Airport, and 3–10 m thick crushed-rock subgrade was used in the Mohe Gulian Airport of China and Yichun Airport of China [8, 9]. Qin and Zhang [10], Qian et al. [11], Liu et al. [12], and Mu et al. [13] discussed the stability of crushed-rock subgrade for highway and railway engineering based on field tests. Because such field tests were characterized by high randomness, long study period, high costs, and topographical and geomorphological limitations, scholars have employed laboratory tests and numerical simulations to further explore the feasibility of crushed-rock subgrade in permafrost regions. Bian et al. [14], Chou et al. [15], and Duan [16] studied the influence of thickness, particle diameter, and boundary effect of crushed-rock on subgrade stability using laboratory tests. Chen et al. [17, 18], Yu et al. [19, 20], Hou [21], and Wang [22] established highway, railway, and airport runway models of the water-heat-force coupling response and analyzed the influence of crushed-rock thickness, particle diameter, and boundary effect to evaluate subgrade stability.

The insulation subgrade method employs insulation materials to reduce heat transfer from the outside to the permafrost to slow permafrost temperature changes and degradation, which is beneficial for maintaining the temperature field stability of the subgrade. The first insulation subgrade was utilized at Kotzebee Airport and Inigok Airport in Alaska, with extruded polystyrene foam used as the insulation material [3]. Both the Qinghai-Tibet Railway and Qinghai-Tibet Highway in China used rigid polyurethane foam and extruded polystyrene foam as insulation materials to maintain temperature field stability of the permafrost subgrade [23–25]. To further analyze the effects of various insulation materials, researchers have conducted indoor experiments and numerical simulation studies and discussed the influence of insulation material, thickness, position, and boundary condition on subgrade stability [26–28].

Crushed-rock and insulation subgrades exhibit many advantages for ensuring permafrost subgrade stability. However, crushed-rock subgrade is not suitable for a wide distribution and thick layer of permafrost owing to the limits of excavation, backfill, earthwork volume, and construction costs. The insulation subgrade method prevents the outward transfer of internal heat, but the permeability, strength, thickness, and durability of the insulation material also affect permafrost subgrade stability. In China, the large airport is called “Hub airport”, which refers to the airport with dense international and domestic air routes, the node of the central air route network, and also the distribution center of air passenger and cargo transport. The main features of the large airport are a high proportion of transit operations and efficient flight connectivity, where passengers can easily transfer to other airports. Hence, the large airport needs to meet 4F-class airport standards, can take off and land the aircraft of A380-800. And the 4F-class airport is an airport with an available runway length of 3600 m or more, a wingspan of 65–80 m for the largest aircraft available, and a distance of 14-16m from the outer wheels of the main landing gear under standard conditions. Therefore, it is necessary to determine the optimal combination of crushed-rock and insulation subgrades in the large airport. This study established and verified a runway model for large airports and analyzed the influence of crushed-rock layer thickness and particle diameter, and insulation layer thickness and location on subgrade stability. Finally, this paper proposed optimization parameters for combined crushed-rock and insulation subgrade in permafrost regions. The research findings provide valuable information informing the design, construction, operation, maintenance, safety, and management of airports in permafrost regions.

## 2. Conditions for solving numerical simulations

### 2.1 Heat conduction equations

The runway is disturbed by solar radiation and precipitation, geological lithology, and the water content of its structural layer and its lower soil layer. Therefore, to optimize the model, improve its computational efficiency, and ensure the effectiveness of the results, the following practical assumptions are made: (1) The layers of the pavement, natural surface, and subgrade are uniform and isotropic. (2) The boundary of each structural layer does not permit moisture migration. (3) The pavement, natural surface, and subgrade layers obey the law of energy conservation and feature only the effects of the heat transfer. Harlan and Taylor propose an equation for the material migration during freezing and thawing. Three-dimensional (3D) unsteady heat conduction can be expressed by Eq (1) [29]:

$$C\frac{\partial T}{\partial t}-L\rho_{i}\frac{\partial w_{i}}{\partial t}=\frac{\partial }{\partial x}(k_{x}\frac{\partial T}{\partial x})+\frac{\partial }{\partial y}(k_{y}\frac{\partial T}{\partial y})+\frac{\partial }{\partial z}(k_{z}\frac{\partial T}{\partial z})$$ (1)

where *C* is the heat capacity, *T* is the temperature, *t* is the time, *L* is the transformed latent heat of water, 334560 J (m)^-3^, *ρ*_*i*_ is the density of ice, 0.917×10^3^ kg (m)^-3^, *w*_*i*_ is the volume fraction of ice, and *k*_*x*_, *k*_*y*_, and *k*_*z*_ are components of thermal conductivity.

During the freezing and thawing of soil, 3D mass transfer can be expressed by Eq (2) when evaporation and other factors are ignored [19]:

$$\frac{\partial w_{u}}{\partial t}+\frac{\rho_{i}}{\rho_{w}}\frac{\partial w_{i}}{\partial t}=\frac{\partial }{\partial x}(K_{x}\frac{\partial \phi }{\partial x})+\frac{\partial }{\partial y}(K_{y}\frac{\partial \phi }{\partial y})+\frac{\partial }{\partial z}(K_{z}\frac{\partial \phi }{\partial z})$$ (2)

where *w*_*u*_ is the volume fraction of unfrozen water; *ρ*_*w*_ is the density of ice, 1×10^3^ kg (m)^-3^; *K*_*x*_, *K*_*y*_, and *K*_*z*_ are the components of hydraulic conductivity; *φ* is the total potential energy, equal to *ϕ* and *h*; *ϕ* is the volumetric potential energy; and *h* is the gravitational potential, which is ignored.

The content of unfrozen water is the mass ratio of unfrozen water to soil particles at a certain negative temperature, as shown in Eq (3):

$$\frac{\partial w_{u}}{\partial t}=\frac{\partial w_{u}}{\partial T}\frac{\partial T}{\partial t}$$ (3)

Eq (4) can be obtained based on Eqs (1), (2), and (3).


$$\left(C+L\rho_{w}\frac{\partial w_{u}}{\partial T})\frac{\partial T}{\partial t}=\frac{\partial }{\partial x}(k_{x}\frac{\partial T}{\partial x})+\frac{\partial }{\partial y}(k_{y}\frac{\partial T}{\partial y})+\frac{\partial }{\partial z}(k_{z}\frac{\partial T}{\partial z})+L\rho_{w}[\frac{\partial }{\partial x}(K_{x}\frac{\partial \phi }{\partial x})+\frac{\partial }{\partial y}(K_{y}\frac{\partial \phi }{\partial y})+\frac{\partial }{\partial z}(K_{z}\frac{\partial \phi }{\partial z})\right]$$ (4)

The volumetric potential of unfrozen water at different temperatures can be determined according to the relationship between temperature and content of unfrozen water and the characteristic curve of water, as shown in Eq (5):

$$\frac{\partial \phi }{\partial t}=\frac{\partial \phi }{\partial w_{u}}\frac{\partial w_{u}}{\partial T}$$ (5)

Eq (6) can be obtained by substituting the differential water capacity and water diffusion coefficient into Eq (5). Eq (7) can be obtained by using Eq (6) in Eq (4).

$$\frac{\partial \phi }{\partial x}=\frac{\partial \phi }{\partial T}\frac{\partial T}{\partial x}=\frac{D}{K}\frac{\partial w_{u}}{\partial T}\frac{\partial T}{\partial x}$$ (6)

$$(C+L\rho_{w}\frac{\partial w_{u}}{\partial T})\frac{\partial T}{\partial t}=\frac{\partial }{\partial x}(k_{x}+L\rho_{w}D_{x}\frac{\partial w_{u}}{\partial T})\frac{\partial T}{\partial x}+\frac{\partial }{\partial y}(k_{y}+L\rho_{w}D_{y}\frac{\partial w_{u}}{\partial T})\frac{\partial T}{\partial y}+\frac{\partial }{\partial z}(k_{z}++L\rho_{w}D_{z}\frac{\partial w_{u}}{\partial T})\frac{\partial T}{\partial z}$$ (7)

where *δ* is the differential capacity of water, *D* is the coefficient of diffusion of water, and *K* is the hydraulic conductivity.

Finally, Eq (7) can be transformed into a solution for 3D nonlinear equations, as shown in Eqs (8)–(12):

$$C(T)\frac{\partial T}{\partial t}=\frac{\partial }{\partial x}(\lambda_{x}(T)\frac{\partial T}{\partial x})+\frac{\partial }{\partial y}(\lambda_{y}(T)\frac{\partial T}{\partial y})+\frac{\partial }{\partial z}(\lambda_{z}(T)\frac{\partial T}{\partial z})$$ (8)

$$C(T)=C+L\rho_{w}\frac{\partial w_{u}}{\partial T}$$ (9)

$$\lambda_{x}(T)=k_{x}+L\rho_{w}D_{x}\frac{\partial w_{u}}{\partial T}$$ (10)

$$\lambda_{y}(T)=k_{y}+L\rho_{w}D_{y}\frac{\partial w_{u}}{\partial T}$$ (11)

$$\lambda_{z}(T)=k_{z}+L\rho_{w}D_{z}\frac{\partial w_{u}}{\partial T}$$ (12)

where *C*(*T*) is the equivalent heat capacity, and *λ*_*x*_(*T*), *λ*_*y*_(*T*), and *λ*_z_(*T*) are the equivalent thermal conductivities.

### 2.2 Boundary conditions

The first boundary condition is established based on the surface temperature of the permafrost region and is applied to the surface in the numerical models, as shown in Eq (13) [18].

$$T=T_{0}+\Delta T+a\sin(\frac{2\pi t}{8760}+b)+\frac{\alpha t}{8760\times 30}$$ (13)

where *T*_0_ is the mean annual ground temperature of the boundary layer, taken as the mean annual air temperature; *ΔT* is the increment in the temperature of the boundary layer, which is set to 2.5°C for the natural surface and 4.5°C for the pavement; *a* is the amplitude of variation in temperature, which is set to 12°C for the natural surface and 15°C for the pavement; *t* is the time in hours; *b* is the initial phase, equal to zero at the initial set time of April 1 in northeast China; and *α* is the increment in the global temperature over the next 30 years, which is set to 1.5°C.

The second boundary condition is an adiabatic boundary condition that is applied on both sides of the numerical models, as shown in Eq (14) [30]:

$$\frac{\partial T}{\partial n}=0$$ (14)

The third boundary condition is a constant temperature gradient of 0.03°C (m). This is applied at the bottom of the numerical models, as shown in Eq (15) [31].


$$\frac{\partial T}{\partial n}=0.03$$ (15)

### 2.3 Parameters of aircraft

**Fig 1:**
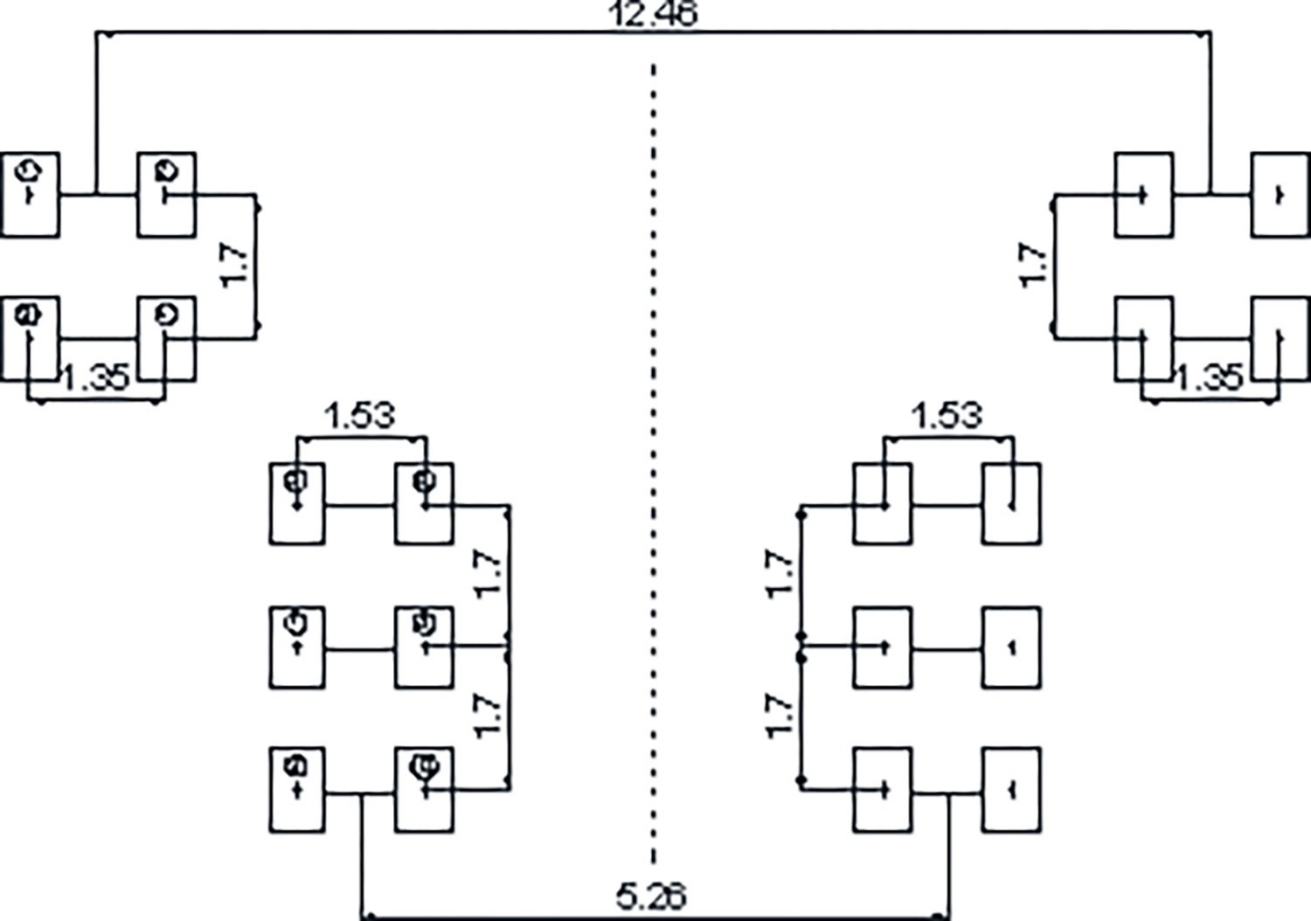
Configuration of the main landing gear of A380-800 (unit: m).

The configuration of the main landing gear is shown in Fig 1, and the aircraft parameters are listed in Table 1 [32].

**Table 1:** Aircraft parameters.

Aircraft type	Maximum take-off weight (kN)	Number of main landing gears	Number of wheels	Main landing gear configuration	Main landing gear space (m)	Wheel space (m)	Wheel load (kN)	Tire pressure (MPa)
A380- 800	5600	4	4/6	Three-axle double wheel	5.26/12.46	1.53/1.35	266	1.47

## 3. Numerical simulations

### 3.1 Numerical model

**Fig 2:**
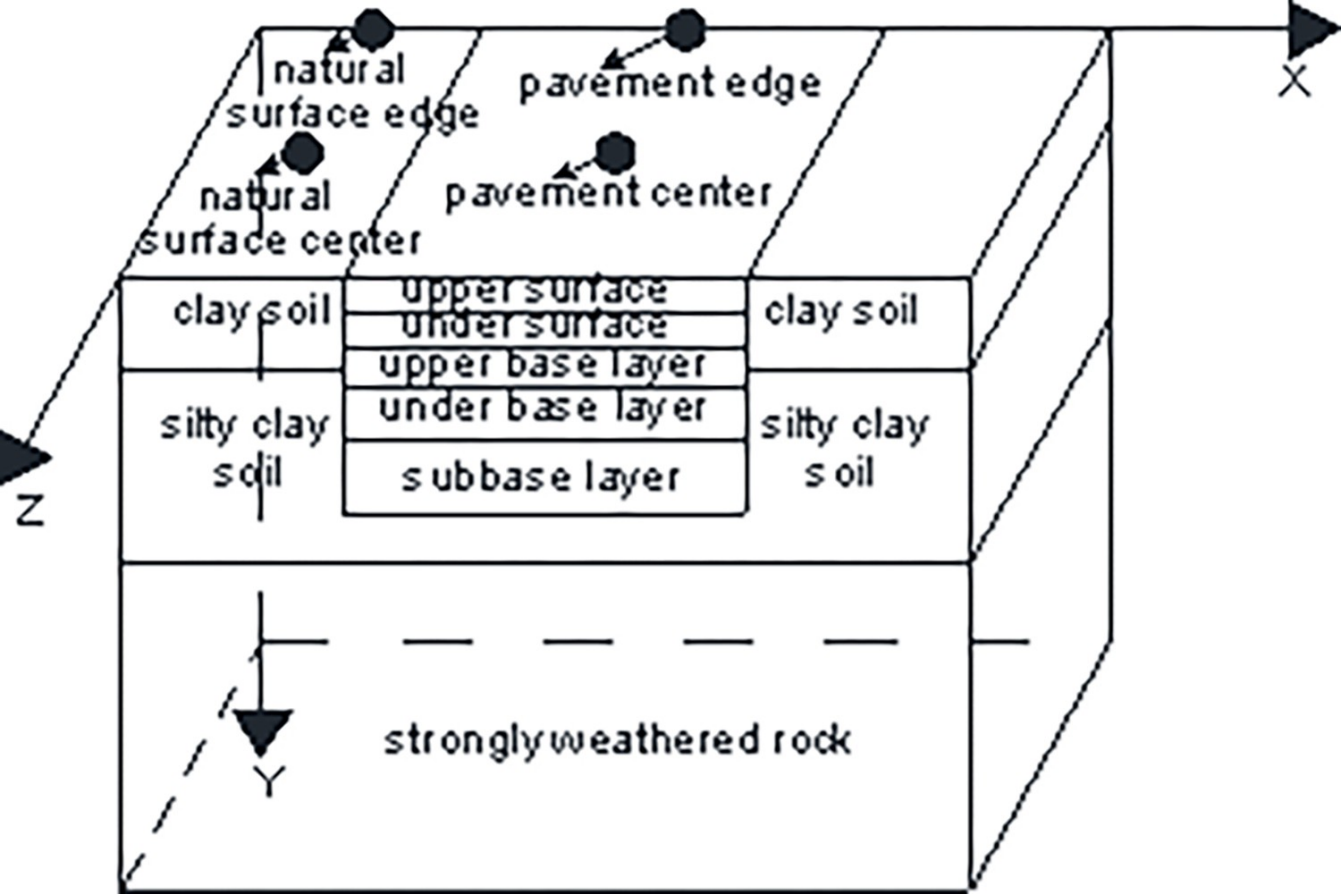
Schematic diagram of numerical model.

As shown in Fig 2, the results of an engineering geological survey indicated that the natural surface consisted of 0.4 m clay soil, 1.6 m silty clay soil, and 18 m strongly weathered rock [33]. According to the design specifications of asphalt pavements for civil airports, the structure of the pavement comprised an upper surface, under surface, upper base layer, under base layer, and subbase layer. To eliminate the boundary effect, a 40 m-wide natural surface was placed on both sides of the pavement structure. For the numerical model, the pavement structure was 45 m wide and 15 m long. Its depth was along the y direction, its width was along the x direction, and its length was along the z direction.

The calculation time was 10 years. The boundary conditions of the numerical model were determined using Eqs (13), (14), and (15). The bottom of the strongly weathered rock layer denied any degree of freedom. The edges of the layers of strongly weathered rock, silty clay soil, and clay soil were constrained along the structure’s length and width. The initial temperature field was set to -1°C [16]. The apparent heat capacity method was used to simulate the phase change that occurred within a certain temperature range. The thermal parameters of the weathered rock, silty clay soil, clay soil, and pavement layers are listed in Tables 2 and 3 [33]. A uniformly distributed rectangular load was applied to simulate the static load of the A380-800 aircraft, arranged symmetrically along the centerline of the upper surface. The Mohr-Coulomb constitutive model was used to characterize the elastoplastic behavior of the strongly weathered rock, silty clay soil, and clay soil. Reference [33] had shown that the mechanical parameters of permafrost were closely related to the temperature, and could be solved by Eqs (16)–(19). The dilatancy angle could be taken as half of the friction angle. The elastic modulus, Poisson’s ratio, cohesion, frictional angle, and dilatancy angle are shown in Table 4. The coefficients of expansion of the strongly weathered rock, silty clay soil, and clay soil layers are listed in Table 5 [33]. The mechanical parameters of the pavement structure layers are listed in Tables 6 and 7 [33].

**Table 2:** Thermal parameters of layers of strongly weathered rock, silty clay soil, and clay soil.

Thermal parameter		-20°C	-10°C	-5°C	-2°C	-1°C	-0.5°C	0°C	20°C
Clay soil	Density (kg (m)^-3^)	1870	1870	1870	1870	1870	1870	1870	1870
	Thermal conductivity (W/(m·°C))	2.4	2.4	2.4	2.4	2.4	2.4	1.54	1.54
	Heat capacity (J/(kg·°C))	835	840	850	860	870	900	1070	1070
Silty clay soil	Density (kg (m)^-3^)	1950	1950	1950	1950	1950	1950	1950	1950
	Thermal conductivity (W/(m·°C))	1.81	1.81	1.81	1.81	1.81	1.81	1.5	1.5
	Heat capacity (J/(kg·°C))	970	1050	1090	1115	1140	1210	1285	1285
Strongly weathered rock	Density (kg (m)^-3^)	2150	2150	2150	2150	2150	2150	2150	2150
	Thermal conductivity (W/(m·°C))	2.5	2.5	2.5	2.5	2.5	2.5	2.01	2.01
	Heat capacity (J/(kg·°C))	950	1060	1110	1140	1190	1250	1350	1350

**Table 3:** Thermal parameters of layers of the pavement structure.

Pavement structure	Material	Density (kg (m)^-3^)	Thickness (m)	Heat capacity (J/(kg·°C))	Thermal conductivity (W/(m·°C))
Upper surface	Asphalt concrete	2300	0.15	1670	1.15
Under surface	Asphalt concrete	2320	0.15	1670	1.20
Upper base layer	Cement-stabilized macadam	2200	0.3	960	1.56
Under base layer	Cement-stabilized macadam	2233	0.35	920	2.04
Subbase layer	Graded macadam	2000	0.5	1100	1.68

**Table 4:** Mechanical parameters of the strongly weathered rock, silty clay soil, and clay soil.

Mechanical parameter		-20°C	-10°C	-5°C	-2°C	-1°C	-0.5°C	0°C	20°C
Clay soil	Elastic modulus (MPa)	177	126	92	64	51	42	26	26
	Poisson’s ratio	0.240	0.320	0.360	0.384	0.392	0.396	0.400	0.400
	Cohesion (MPa)	1.960	1.060	0.610	0.340	0.250	0.205	0.160	0.160
	Frictional angle (/°)	27.00	21.00	18.00	16.20	15.60	15.30	15.00	15.00
	Dilatancy angle (/°)	13.50	10.50	9.00	8.10	7.80	7.65	7.50	7.50
Silty clay soil	Elastic modulus (MPa)	191	135	99	69	55	46	28	28
	Poisson’s ratio	0.210	0.280	0.315	0.336	0.343	0.347	0.350	0.350
	Cohesion (MPa)	1.950	1.050	0.600	0.330	0.240	0.195	0.150	0.150
	Frictional angle (/°)	26.00	20.00	17.00	15.20	14.60	14.30	14.00	14.00
	Dilatancy angle (/°)	13.00	10.00	8.50	7.60	7.30	7.15	7.00	7.00
Strongly weathered rock	Elastic modulus (MPa)	199	141	104	72	58	48	30	30
	Poisson’s ratio	0.180	0.240	0.270	0.288	0.294	0.297	0.300	0.300
	Cohesion (MPa)	1.945	1.045	0.595	0.325	0.235	0.190	0.145	0.145
	Frictional angle (/°)	24.50	19.00	16.25	14.60	14.05	13.78	13.50	13.50
	Dilatancy angle (/°)	12.25	9.50	8.13	7.30	7.03	6.89	6.75	6.75

**Table 5:** Expansion coefficients of layers of strongly weathered rock, silty clay soil, and clay soil.

Temperature (°C)	Clay soil	Silty clay soil	Strongly weathered rock
-30	-5.33×10^−5^	-6.08×10^−5^	-8.55×10^−5^
-20	-1.22×10^−4^	-1.38×10^−4^	-1.22×10^−4^
-18	-1.39×10^−4^	-1.61×10^−4^	-1.43×10^−4^
-10	-3.26×10^−4^	-3.66×10^−4^	-3.18×10^−4^
-5	-1.38×10^−3^	-2.04×10^−3^	-1.26×10^−3^

**Table 6:** Mechanical parameters of pavement structure layers.

Pavement structure	Material	Elastic modulus (MPa)	Poisson’s ratio	Expansion coefficient
Upper surface	Asphalt concrete	See Table 7	0.25	$\beta =2.496e^{0.01467T}\times 10^{-5}$
Under surface	Asphalt concrete	See Table 7	0.25	$\beta =2.496e^{0.01467T}\times 10^{-5}$
Upper base layer	Cement-stabilized macadam	3800	0.25	3.80×10^−5^
Under base layer	Cement-stabilized macadam	3400	0.25	3.40×10^−5^
Subbase layer	Graded macadam	175	0.35	1.75×10^−4^

*β* is the expansion coefficient.

**Table 7:** Elastic moduli of the surface layer.

Temperature (°C)	-30	-20	-10	0	10	20	30
Elastic modulus (MPa)	8046	5011	3160	1986	1021	784	452


$$E=a_{1}+b_{1}{\left|T\right|}^{m}$$ (16)

$$\upsilon =a_{2}+b_{2}|T|$$ (17)

$$c=a_{3}+b_{3}|T|$$ (18)

$$\theta =a_{4}+b_{4}|T|$$ (19)

where *E* is the elastic modulus; *ν* is the Poisson’s ratio; *c* is the cohesion; *θ* is the friction angle; and *a*_1_, *a*_2_, *a*_3_, *a*_4_, *b*_1_, *b*_2_, *b*_3_, and *b*_4_ are the regression coefficients. When *T* is not less than zero, *b*_1_, *b*_2_, *b*_3_, and *b*_4_ are all equal to zero. *m* is the index, which is set at 0.6.

### 3.2 Verifying the numerical model

**Fig 3:**
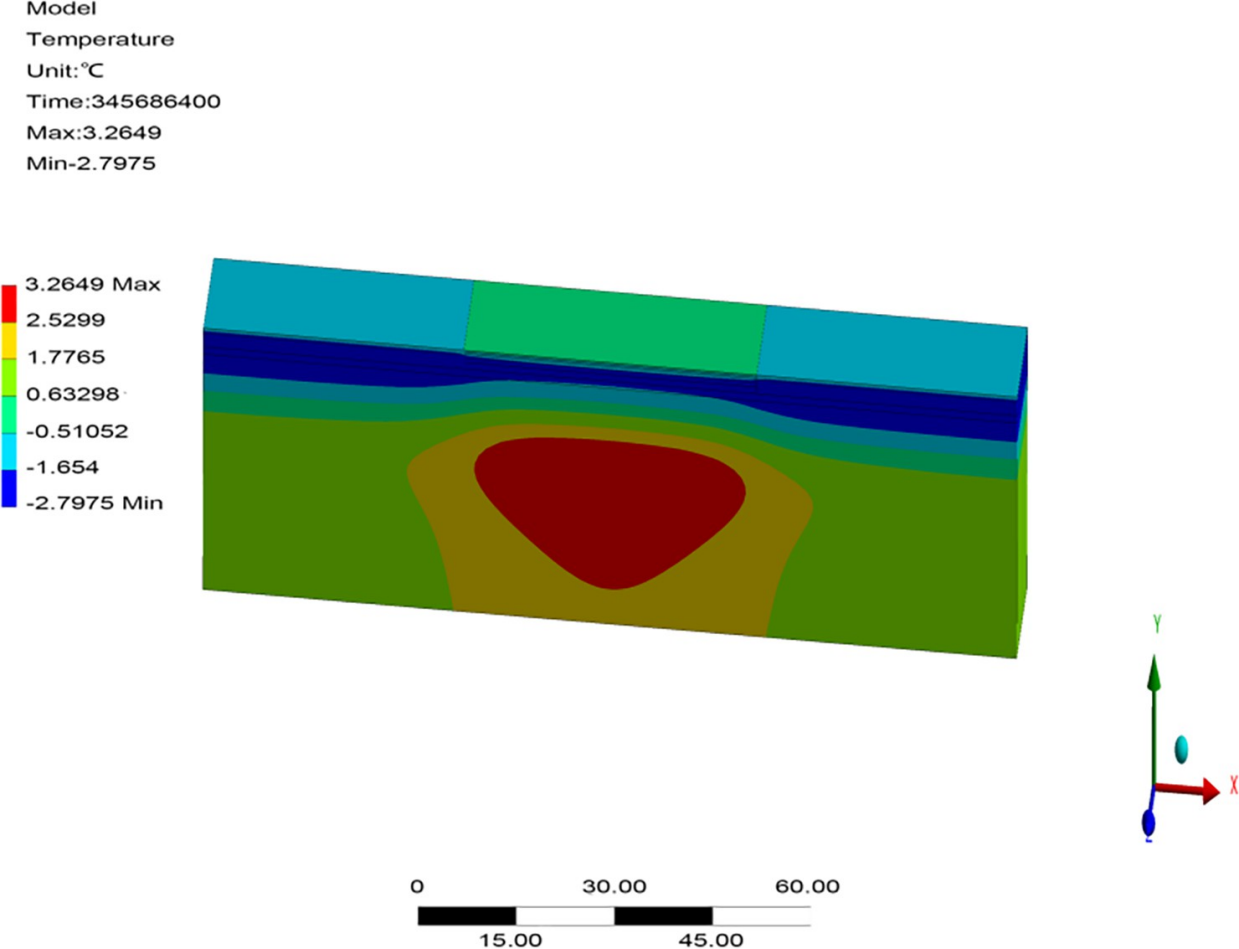
Temperature field after 10 years of operation in the northeast of China.

**Fig 4:**
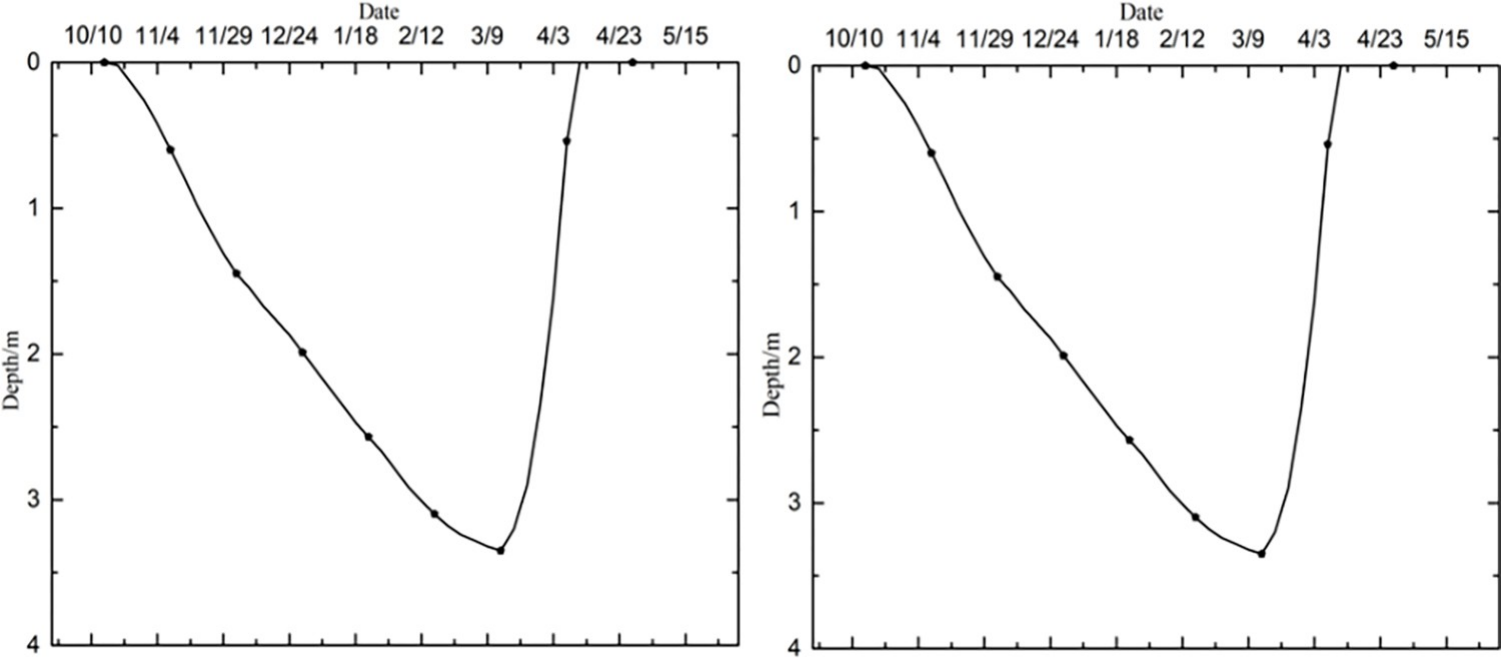
Depth of the frozen layer after 10 years of operation at (a) the center and (b) edge of the pavement.

**Fig 5:**
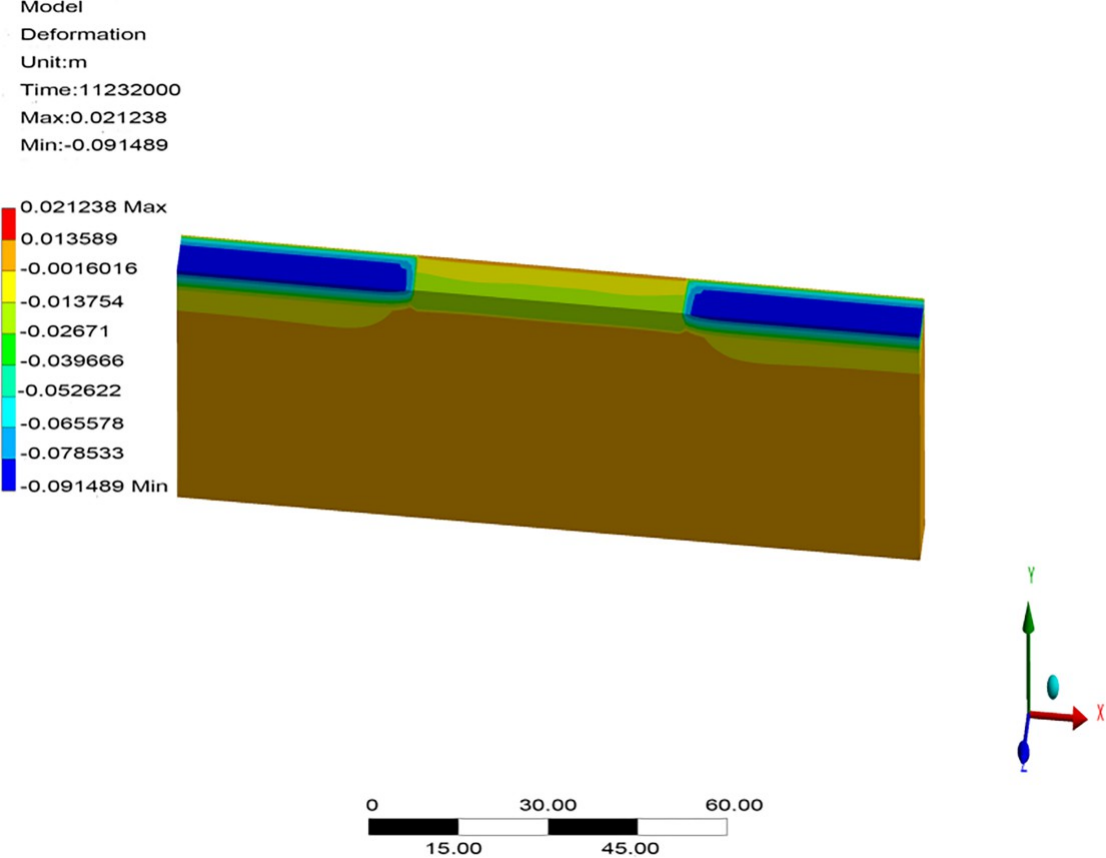
Displacement field after one year of operation in the northeast of China.

Owing to a lack of monitoring data on runways, the reliability of the numerical model was verified through a comparison with previously proposed models in terms of the temperature field, frozen layer depth, and settlement without considering aircraft load. Transient thermal analysis of the runway was conducted over 10 years based on the initial temperature field. The temperature field after 10 years of operation in northeast China is shown in Fig 3. The change in the depth of the frozen layer with respect to time after 10 years of operation is shown in Fig 4. The displacement field after one year of operation is shown in Fig 5.

Fig 3 showed that the maximum depth of the frozen pavement layer was obtained on the 346^th^ day, after the air temperature had reached its maximum value at the end of March in the second year. The appearance time of the maximum depth of the frozen pavement layer in Fig 3 accorded with those reported by Liu et al. [12] and Wang [34]. Fig 4 showed that the pavement began to freeze in the middle of October, and its frozen layer reached its maximum depth at the end of March. It began to thaw at the beginning of April and had thawed completely by the end of April. The freeze-thaw characteristics and temporal regularity of the pavement in Fig 4 were consistent with those reported by Li [33] and Duan [16]. However, the maximum depth of the frozen pavement layer in runway engineering was about 3.35 m, deeper than 2.67–3.05 m in highway and railway engineering. The maximum depth of the frozen layer in Fig 4 was consistent with 3.15–3.65 m reported by Bjella [35] as well as by Simon and Dilley [36]. Fig 5 showed that the maximum settlement of the pavement occurred on the 40^th^ day after the air temperature had reached its maximum value in the middle of August. Maximum settlement was observed for the pavement and natural surface at 0.023 m and 0.098 m, respectively, in runway engineering. The maximum settlement characteristics and temporal regularity in Fig 5 were in accord with those reported by LeBlanc et al. [37]. Therefore, the parameters and boundary conditions of the model of the runway structure were reasonable, indicating its applicability for analyzing the response of permafrost runways to settlement.

## 4. Results and discussion

### 4.1 Changes in temperature

**Fig 6:**
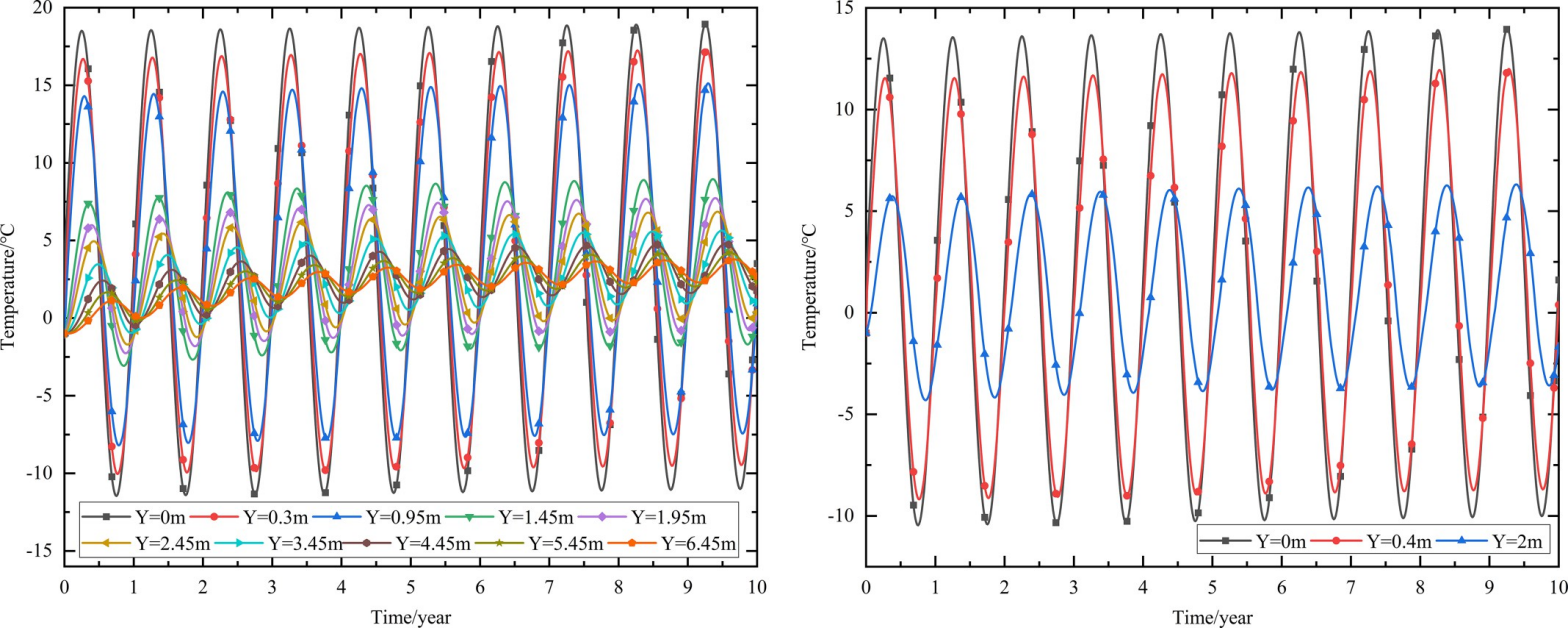
Temperature-time curves at the (a) pavement center and (b) natural surface center.

**Fig 7:**
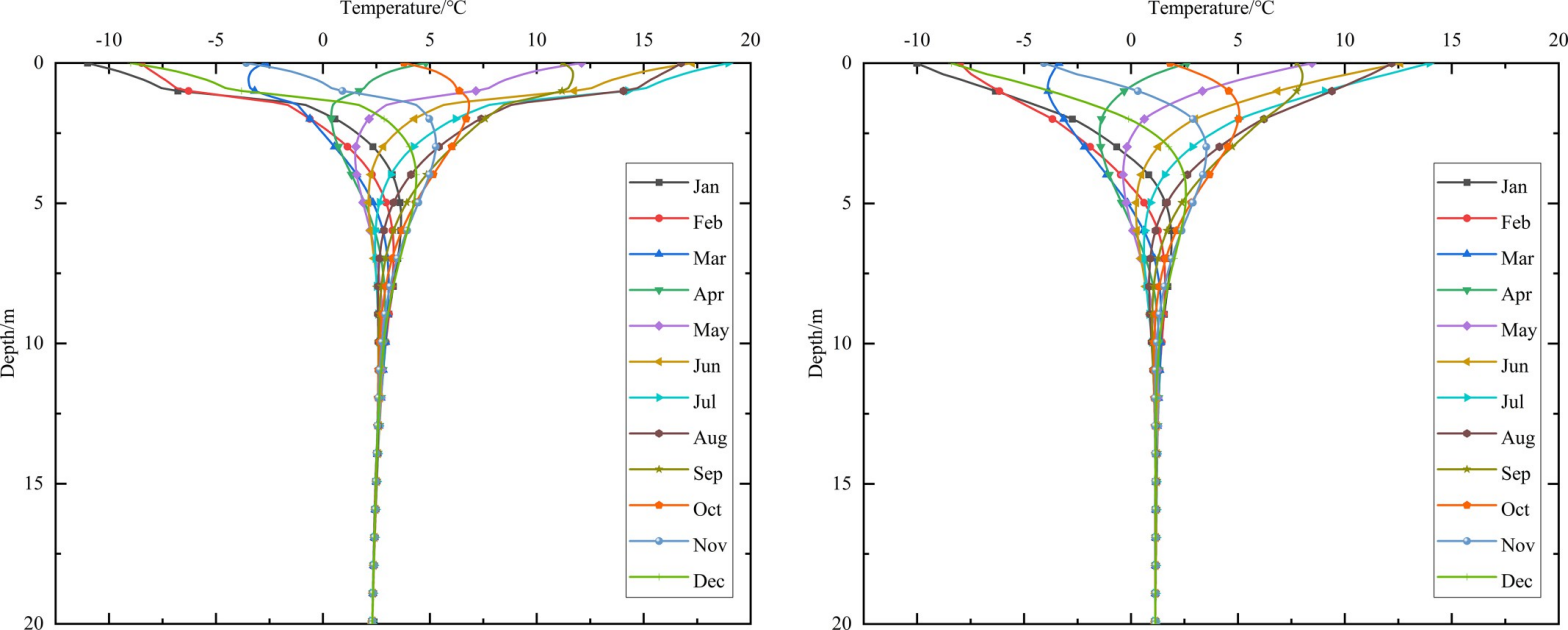
Temperature-depth curves at the (a) pavement center and (b) natural surface center.

As shown in Fig 2, the thickness of the subbase layer remains the same, and the particle size, density, and thermal parameters of the subbase layer are varied, as shown in Table 8. The parameters of the installation layer are listed in Table 9 [34]. When the 0.10 m-thickness installation layer is at the bottom of the subbase layer and the 0.50 m-thickness subbase layer is the particle size of 4–6 cm, the time-dependent temperature curves are shown in Fig 6 and the depth-dependent temperature curves are shown in Fig 7.

**Table 8:** Thermal parameters of varied subbase layer.

Material	Particle size (cm)	Density (kg (m)^-3^)	Thermal conductivity (W/(m·°C))	Heat capacity (J/(kg·°C))
Graded crushed-rock 1	4–6	1500	0.407	840
Graded crushed-rock 2	6–8	1490	0.385	838.9
Graded crushed-rock 3	10–15	1410	0.345	839.7

**Table 9:** Parameters of installation layer.

Material	Thickness (m)	Density (kg (m)^-3^)	Thermal conductivity (W/(m·°C))	Heat capacity (J/(kg·°C))
XPS	0.10,0.15,0.20	30	0.030	1250

As shown in Fig 6, as depth increased, the temperature amplitudes of the pavement and natural surface gradually decreased with phase delay. This phenomenon occurred because much of the heat in the pavement did not dissipate quickly and required a long time to gradually dissipate. Moreover, the temperature gradually decreased as the heat conduction depth increased. Finally, the pavement and natural surface reached a new thermal dynamic equilibrium after five or six years. In addition, the temperatures of the pavement and natural surface exhibited a slight rising trend owing to the impact of global climate change.

Fig 6(A) and 6(B) indicated that the temperature of the pavement was higher than that of the natural surface at the same depth and time. The difference in the highest temperature of the pavement and that of the natural surface was approximately 5°C, whereas the difference in the lowest temperature for each was approximately 1°C. The phase delay in the runway was greater than that in highways and railways. This was because both the solar absorption coefficient and the quantity of heat absorbed by the pavement were greater than those of the natural surface. Furthermore, the runway was wider than a highway or railway. Therefore, heat absorption area was larger, making the quantity of heat absorbed by the runway greater than that absorbed by the highway or railway and leading to a longer time required for heat dissipation.

As shown in Fig 6(A), when the depth was 0.95 m (bottom of the under base layer), the temperature of the pavement varied from -8.2°C to 15.1°C. When the depth was 1.45 m (bottom of the subbase layer), the temperature of the pavement varied from -3.1°C to 9.0°C. Therefore, the subbase layer of the graded crushed-rock could significantly reduce the temperature range of the time-dependent curve and was beneficial for maintaining the temperature stability of the subgrade.

As shown in Fig 7, the temperature envelopes of the pavement and natural surface remained funnel-shaped over the year. The temperature ranges of the pavement and natural surface decreased as depth increased and tended to be constant. The upper temperature-depth curves of the pavement and natural surface that were shifted to the left indicated that low-temperature interlayers existed in the pavement structure and natural surface. Similarly, the upper temperature-depth curves that were shifted to the right indicated that high-temperature interlayers existed in the pavement structure and natural surface. These temperature characteristics were similar to those observed in highway and railway engineering [28, 34]. As shown in Fig 7(A), when the depth was 1.45 m (bottom of the subbase layer), the temperature of the pavement showed a steep decline. This phenomenon was similar to that shown in Fig 6(A) and also indicated that the subbase layer of the graded crushed-rock was suitable for reducing the temperature range of the depth-dependent curve to maintain temperature stability in the subgrade.

When the particle sizes of the subbase layers were 6–8 cm and 10–15 cm, the evolution laws of temperature-time and temperature-depth curves were similar to those shown in Figs 6 and 7, respectively. Hence, the data presented in Table 10 were used to further compare the temperature range when the particle size of the subbase layer varied from 4–6 cm to 10–15 cm.

**Table 10:** Temperature of varied subbase layer.

Parameter	Location	Value		
Particle size (cm)	Pavement center	4–6	6–8	10–15
Thickness (m)	Pavement center	0.5	0.5	0.5
Temperature of 0 m (°C)	Pavement center	-11.46–18.95	-11.46–18.95	-11.46–18.95
Temperature of 0.3 m (°C)	Pavement center	-10.04–17.31	-10.06–17.32	-10.08–17.34
Temperature of 0.4 m (°C)	Natural surface center	-9.19–12.00	-9.19–12.00	-9.19–12.00
Temperature of 0.95 m (°C)	Pavement center	-8.20–15.13	-8.24–15.17	-8.29–15.22
Temperature of 1.45 m (°C)	Pavement center	-3.07–8.96	-3.03–8.89	-2.99–8.82
Temperature of 1.95 m (°C)	Pavement center	-2.26–7.74	-2.23–7.68	-2.20–7.63
Temperature of 2 m (°C)	Natural surface center	-4.31–6.32	-4.31–6.32	-4.31–6.32
Temperature of 2.45 m (°C)	Pavement center	-1.70–6.86	-1.68–6.81	-1.66–6.77
Temperature of 3.45 m (°C)	Pavement center	-1.0–5.63	-1.0–5.60	-1.0–5.57
Temperature of 4.45 m (°C)	Pavement center	-1.0–4.80	-1.0–4.78	-1.0–4.76
Temperature of 5.45 m (°C)	Pavement center	-1.0–4.21	-1.0–4.19	-1.0–4.18
Temperature of 6.45 m (°C)	Pavement center	-1.0–3.79	-1.0–3.77	-1.0–3.76

Table 10 showed that the particle size of the subbase layer had almost no effect on the temperature of the natural surface center. When the particle size of the subbase layer ranged from 4–6 cm to 10–15 cm, the temperature range of the pavement center decreased at the depth of 0.3–6.45 m. This occurred because the graded crushed-rock hindered the downward transfer of heat from the pavement structure and maintained the temperature stability of the subgrade. Moreover, when the thickness of the subbase layer was the same, the cooling effect of the graded crushed-rock slightly increased with increasing particle diameter of the graded crushed-rock. However, when the particle size of the subbase layer increased from 6–8 cm to 10–15 cm, the porosity and cooling effect of the subbase layer increased slightly with the growth of the particle size of the subbase layer. And the particle size of 10–15 cm was not suitable for construction and leveling in runway engineering. Therefore, the optimization particle size of the subbase layer was 6–8 cm, which was beneficial to maintain the temperature stability of the subgrade and was convenient for the construction of runway engineering.

**Fig 8:**
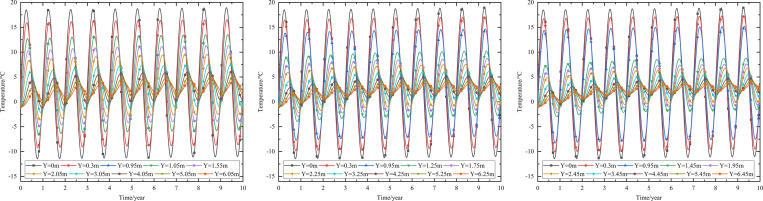
Temperature-time curves of pavement center at the subbase layer thickness of (a) 0.10 m, (b) 0.30 m, and (c) 0.50 m.

When the 0.10 m-thickness installation layer is at the bottom of the subbase layer and the particle size of the subbase layer is 6–8 cm, the time-dependent temperature curves at different thickness of the subbase layer are shown in Fig 8.

Fig 8 showed that the temperature range of the pavement center was similar at the top of the subbase layer. When the subbase layer thickness was 0.10 m, the temperature at the depth of 0.95 m (bottom of the under base layer) was almost the same as that at the depth of 1.05 m (bottom of the subbase layer). When the subbase layer thickness exceeded 0.10 m, the temperature at the bottom of the subbase layer was significantly less than the temperature at the bottom of the under base layer. Hence, the thin graded crushed-rock had little effect on the temperature stability of the subgrade.

Both the temperature and temperature range of the pavement center decreased as subbase layer thickness increased. When the subbase layer thickness varied from 0.10 m to 0.50 m, the maximum temperature at the bottom of the subbase layer decreased by 4.7°C, and the minimum temperature at the bottom of the subbase layer decreased by 3.5°C. As the subbase layer thickness increased from 0.10 m to 0.50 m, the temperature amplitude below the subbase layer varied by decreasing amounts as depth increased. When the depth was 5 m below the subgrade, the maximum and minimum temperatures changed by 0.6°C and 0°C respectively as the subbase layer thickness increased from 0.10 m to 0.50 m. Hence, the increase in the thickness of the graded crushed-rock maintained a reduced temperature of the subgrade, and the cooling effect of the graded crushed-rock gradually diminished as depth increased. Hence, the optimization thickness of the subbase layer was 0.50 m, which was beneficial to maintain the temperature stability of the subgrade and ensure the settlement of replacement construction.

**Fig 9:**
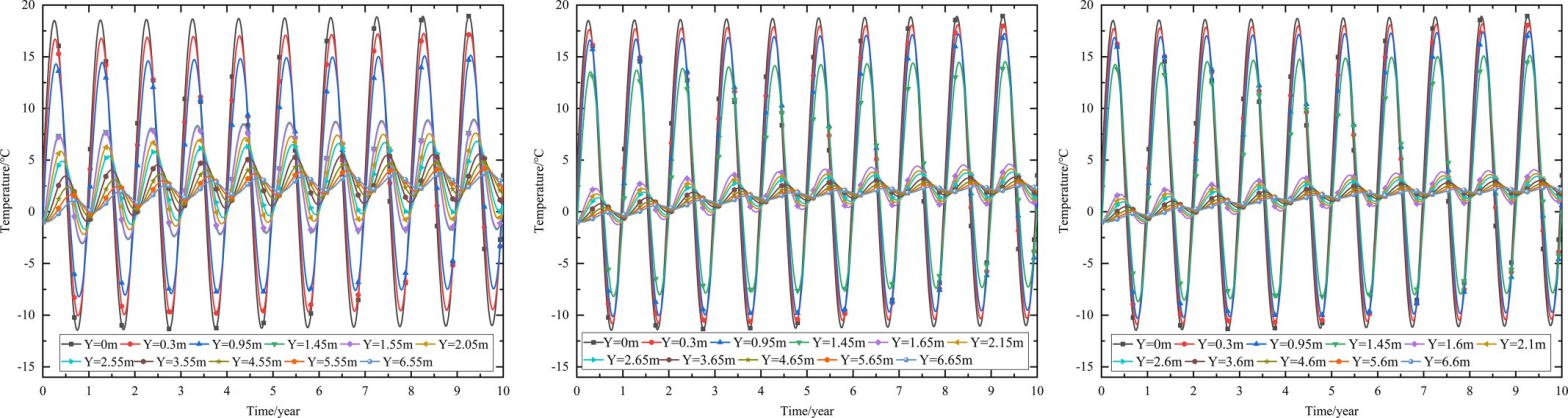
Temperature-time curves of pavement center at the installation layer thickness of (a) 0.10 m, (b) 0.15 m, and (c) 0.20 m.

When the installation layer is at the bottom of the subbase layer and the 0.50 m-thickness subbase layer is the particle size of 6–8 cm, the time-dependent temperature curves at different thickness of installation layer are shown in Fig 9.

Fig 9(A) showed that when the installation layer thickness was 0.10 m, the temperature at the depth of 1.45 m (bottom of the subbase layer) was almost the same as that at the depth of 1.55 m (bottom of the installation layer). Fig 9(B) showed that when the installation layer thickness was 0.15 m, the temperature at the depth of 1.45 m (bottom of the subbase layer) was significantly more than the temperature at the depth of 1.60 m (bottom of the installation layer). Fig 9(C) showed that when the installation layer thickness was 0.20 m, the time-dependent temperature curve was similar to that of the installation layer thickness of 0.15 m. The was because the installation layer prevented the transmission of heat. Thus, the temperature at the top of the installation layer was similar to the temperature of the subbase layer, whereas the temperature at the bottom of the installation layer was close to the temperature of the subgrade. Therefore, the optimization installation layer thickness was 0.15 m, which was beneficial to maintain the temperature stability of the subgrade and economy in manpower and material resources.

**Fig 10:**
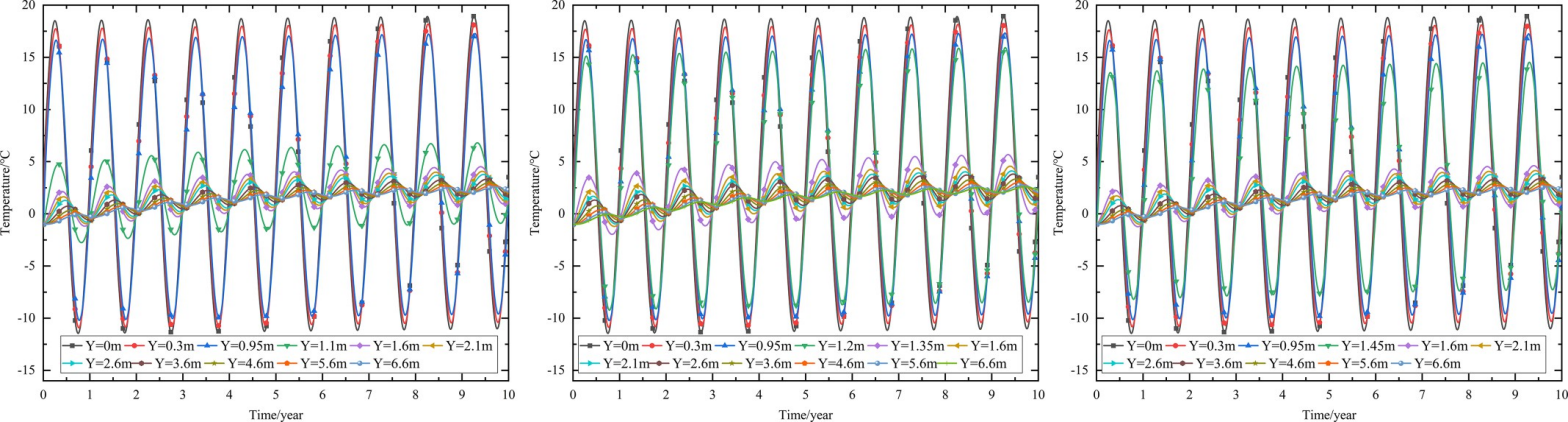
Temperature-time curves of pavement center with the installation layer at (a) the top of the subbase layer, (b) the middle of the subbase layer, and (c) the bottom of the subbase layer.

When the thickness of installation layer is 0.15 m and the 0.50 m-thickness subbase layer is the particle size of 6–8 cm, the time-dependent temperature curves at different location of installation layer are shown in Fig 10.

Fig 10(A) showed that when the installation layer was at the top of the subbase layer, the temperature at the depth of 1.1 m (bottom of the installation layer) was significantly lower than that at the depth of 0.95 m (top of the installation layer), and was about 2.5°C higher than that at the depth of 1.6 m (bottom of the subbase layer). Fig 10(B) showed that when the installation layer was at the middle of the subbase layer, the temperature at the depth of 1.35 m (bottom of the installation layer) was significantly less than the temperature at the depth of 1.2 m (top of the installation layer), and was about 1.25°C higher than that at the depth of 1.6 m (bottom of the subbase layer). Fig 10(C) showed that when the installation layer was at the bottom of the subbase layer, the temperature at the depth of 1.6 m (bottom of the installation layer) was significantly less than the temperature at the depth of 1.45 m (top of the installation layer). Fig 10 showed that the location of installation layer had significant influence on the temperature of the subbase layer, but had little influence on the temperature of the subgrade. The was because the installation layer prevented the transmission of heat, and the temperature at the bottom of the installation layer was close to the temperature of the subbase layer or subgrade. Hence, the optimization location of installation layer was at the bottom of the subbase layer, which was beneficial to maintain the temperature stability of the subgrade and improve the depth of the frozen layer.

### 4.2 Changes in settlement

**Fig 11:**
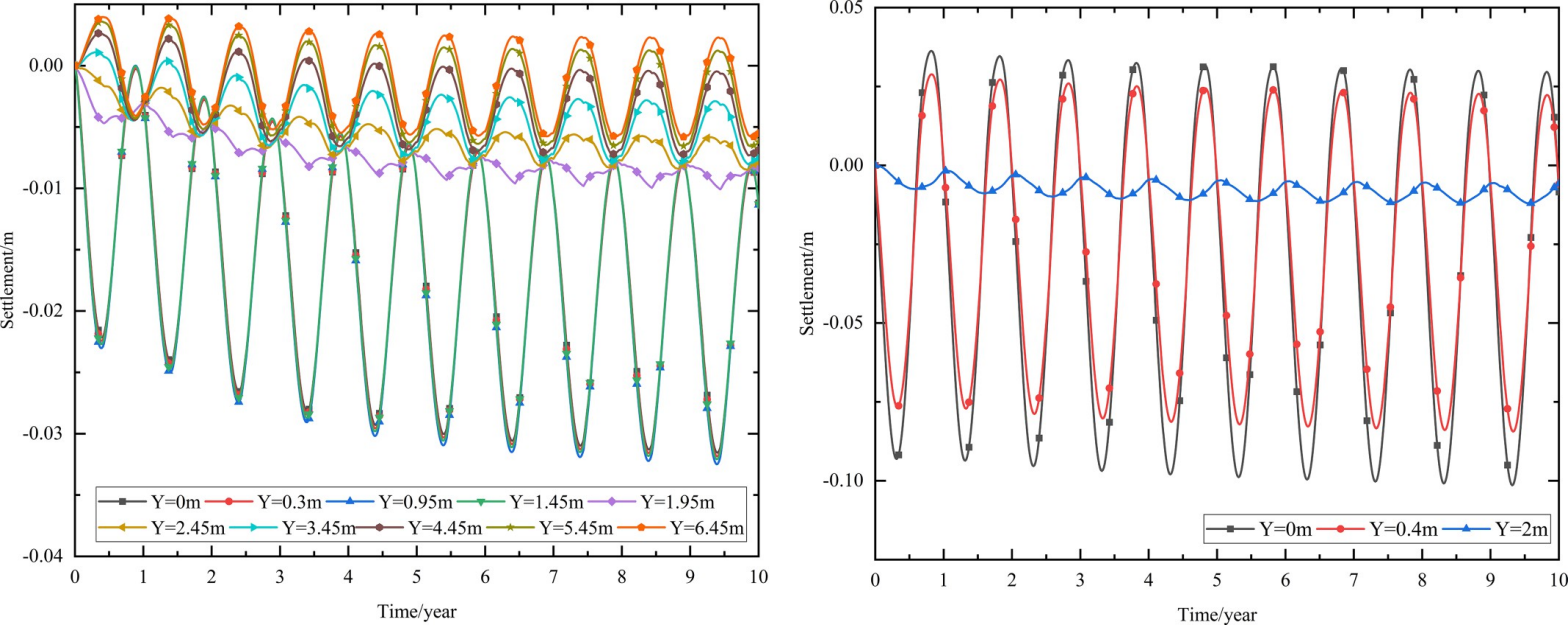
Settlement-time curves at the (a) pavement center and (b) natural surface center.

**Fig 12:**
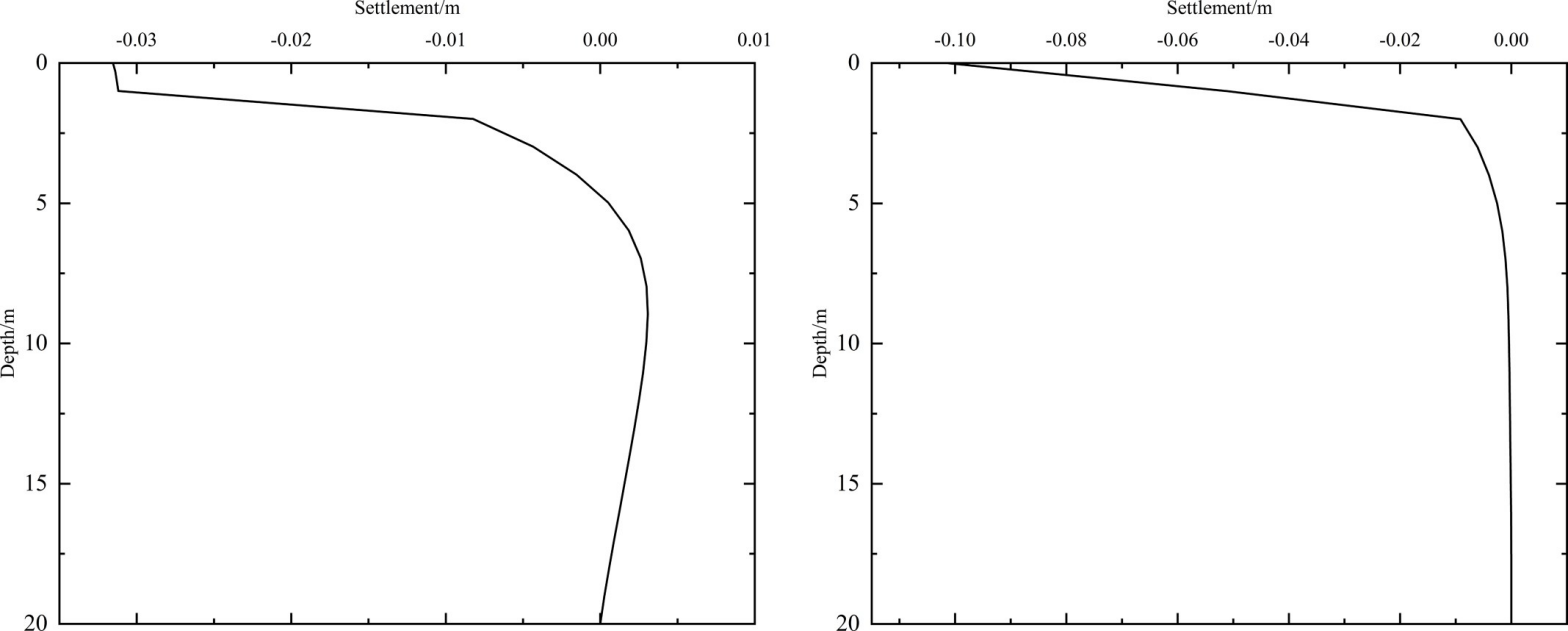
Settlement-depth curves after 10 years of operation at the (a) pavement center and (b) natural surface center.

As shown in Fig 2, the thickness of the subbase layer remains the same, and the particle size, density, and thermal parameters of the subbase layer are varied, as shown in Table 8. The parameters of the installation layer are listed in Table 9 [34]. When the 0.10 m-thickness installation layer is at the bottom of the subbase layer and the 0.50 m-thickness subbase layer is the particle size of 4–6 cm, the time-dependent settlement curves are shown in Fig 11 and the depth-dependent settlement curves are shown in Fig 12.

As shown in Fig 11, as the depth increased, the settlement amplitudes of the pavement and the natural surface gradually decreased with phase delay. This phenomenon occurred because much of the settlement in the pavement did not dissipate quickly and required a long time to develop gradually. Moreover, settlement gradually decreased as the depth increased. Finally, the settlement amplitudes of the pavement and the natural surface gradually decreased over time. Over the first five years, the settlement of the pavement center ranged from -0.023 m to -0.030 m, and the average annual amplitude of the settlement was 0.14 cm/year. However, the settlement of the natural surface ranged from -0.092 m to -0.100 m in the first five years. Subsequently, the pavement and natural surface reached a new settlement dynamic equilibrium after five or six years. This occurred because the temperature of the subgrade increased rapidly in the five years after the pavement structure was built. In this stage, pavement settlement occurred primarily due to the thermal disturbance of the pavement structure. Therefore, the low-temperature permafrost under the subgrade turned into permafrost at high temperatures with high ice content. The instability of this permafrost accelerated settlement. Five years later, the influence of external temperature on the natural surface became evident, and the high-temperature permafrost was in the stage of slow, linear warming. Owing to the continual melting of part of the permafrost, deformation did not continue. Therefore, the settlement of the subgrade gradually decreased.

Fig 11(A) and 11(B) indicated that pavement settlement was higher than that of the natural surface at the same depth and time. The difference in the highest settlement values for the pavement and natural surface was approximately 0.068 m, whereas the difference in the lowest temperatures for each was approximately 0.025 m. As shown in Fig 11(A), when the depth was 0.95 m (bottom of the under base layer), pavement settlement varied from -0.032 m to 0 m. When the depth was 1.45 m (bottom of the subbase layer), pavement settlement varied from -0.01 m to 0 m. Therefore, the subbase layer of the graded crushed-rock significantly reduced the settlement range of the time-dependent curve and was beneficial for maintaining the settlement stability of the subgrade. When the depth was 3.45 m (2 m below the subgrade), pavement settlement had a positive value, indicating that frost deformation occurred. Moreover, frost deformation became increasingly obvious, and the positive value of the settlement increased as depth increased.

In Fig 12, the ranges of the maximum settlement at the pavement and natural surface decreased as depth increased and tended to 0 m. In Fig 12(A), the settlement of the pavement center was small when the depth varied from 0 m to 1.45 m (bottom of the subbase layer) and then increased when the depth was in 1.45 m to 2 m. This occurred because the settlement mainly appeared in the subgrade instead of the pavement structure. When the depth ranged from 5.5 m to 20 m, the settlement of the pavement had a positive value of 0–0.004 m. This phenomenon was similar to that shown in Fig 11(A) and also indicated that the subbase layer of the graded crushed-rock effectively reduced the settlement range of the depth-dependent curve to maintain the settlement stability of the subgrade. Fig 12(A) also showed that the total settlement after 10 years of operation was -0.032 m. The settlement at a depth of 2 m was -0.025 m, accounting for 78% of the total settlement. In Fig 12(B), the settlement of the natural surface center mainly occurred in the range of 0–2 m, which was consisted of clay soil at high temperatures with high ice content and high melting-induced compressibility. The total settlement after 10 years of operation was -0.100 m. The settlement at a depth of 2 m was -0.091 m, which accounted for 91% of the total settlement.

When the particle sizes of the subbase layer were 6–8 cm and 10–15 cm, the evolution laws of the settlement-time and settlement-depth curves were similar to those shown in Figs 11 and 12, respectively. Hence, Table 11 was applied to further compare the settlement range when the particle size of the subbase layer varied from 4–6 cm to 10–15 cm.

**Table 11:** Settlement of varied subbase layer.

Parameter	Location	Value		
Particle size (cm)	Pavement center	4–6	6–8	10–15
Thickness (m)	Pavement center	0.5	0.5	0.5
Settlement of 0 m (m)	Pavement center	-0.0315–0	-0.0310–0	-0.0306–0
Settlement of 0.3 m (m)	Pavement center	-0.0318–0	-0.0313–0	-0.0309–0
Settlement of 0.4 m (m)	Natural surface center	-0.0844–0.0289	-0.0844–0.0289	-0.0844–0.0289
Settlement of 0.95 m (m)	Pavement center	-0.0324–0	-0.0320–0	-0.0315–0
Settlement of 1.45 m (m)	Pavement center	-0.0321–0	-0.0315–0	-0.0311–0
Settlement of 1.95 m (m)	Pavement center	-0.0101–0	-0.0097–0	-0.0094–0
Settlement of 2 m (m)	Natural surface center	-0.0120–0	-0.0120–0	-0.0120–0
Settlement of 2.45 m (m)	Pavement center	-0.0085–0	-0.0083–0	-0.0082–0
Settlement of 3.45 m (m)	Pavement center	-0.0080–0.0011	-0.0079–0.0012	-0.0078–0.0013
Settlement of 4.45 m (m)	Pavement center	-0.0073–0.0027	-0.0072–0.0028	-0.0071–0.0029
Settlement of 5.45 m (m)	Pavement center	-0.0066–0.0036	-0.0065–0.0037	-0.0064–0.0038
Settlement of 6.45 m (m)	Pavement center	-0.0058–0.0039	-0.0057–0.0040	-0.0056–0.0041

Table 11 showed that the particle size of the subbase layer had almost no effect on the settlement of the natural surface center. When the particle size of the subbase layer ranged from 4–6 cm to 10–15 cm, the settlement range of the pavement center increased at the depth of 0.3–0.95 m and decreased at the depth of 1.4–6.45 m. This occurred because the graded crushed-rock was beneficial for maintaining the settlement stability of the subgrade. Moreover, when the depth was the same, the settlement range of the pavement center decreased as the particle size of the graded crushed-rock increased. When the depth exceeded 2.45 m, pavement settlement had a positive value, which might have been caused by frost deformation. However, when the particle size of the subbase layer increased from 6–8 cm to 10–15 cm, the porosity and cooling effect of the subbase layer increased slightly with the growth of the particle size of the subbase layer. And the particle size of 10–15 cm was not suitable for construction and leveling in runway engineering. Therefore, the optimization particle size of the subbase layer was 6–8 cm, which was beneficial to maintain the settlement stability of the subgrade and was convenient for the construction of runway engineering.

**Fig 13:**
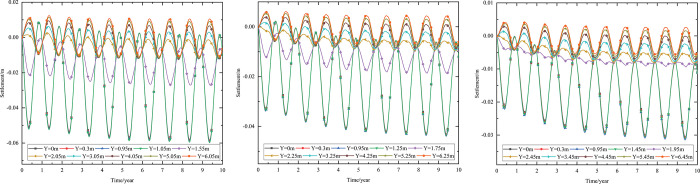
Settlement-time curves of pavement center with subbase layer thickness of (a) 0.10 m, (b) 0.30 m, and (c) 0.50 m.

When the 0.10 m-thickness installation layer is at the bottom of the subbase layer and the particle size of the subbase layer is 6–8 cm, the time-dependent settlement curves at different thickness of the subbase layer are shown in Fig 13.

Fig 13 represented that the settlement decreased as subbase layer thickness increased. Because the increase of the subbase layer thickness was beneficial to maintain the temperature stability of the subgrade and reduce pavement settlement. Moreover, when the subbase layer thickness ranged from 0.10 m to 0.30 m, the positive value of the settlement occurred from 3.05 m (2 m below the subgrade) to 4.25 m (3 m below the subgrade). When the subbase layer thickness ranged from 0.30 m to 0.50 m, the positive value of the settlement occurred from 4.25 m (3 m below the subgrade) to 5.45 m (4 m below the subgrade). This phenomenon also indicated that increasing the subbase layer thickness reduced settlement and maintained subgrade stability. Hence, the optimization thickness of the subbase layer was 0.5 m, which was beneficial to maintain the settlement stability of the subgrade and ensure the settlement of replacement construction.

**Fig 14:**
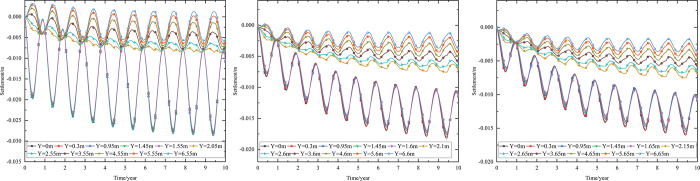
Settlement-time curves of pavement center at the installation layer thickness of (a) 0.10 m, (b) 0.15 m, and (c) 0.20 m.

When the installation layer is at the bottom of the subbase layer and the 0.50 m-thickness subbase layer is the particle size of 6–8 cm, the time-dependent settlement curves at different thickness of installation layer are shown in Fig 14.

Fig 14 represented that the settlement decreased as installation layer thickness increased. Because the increase of the installation layer thickness was beneficial to prevent the transmission of heat and reduce pavement settlement. Moreover, Fig 14(A) showed that when the installation layer thickness was 0.10 m, the positive value of the settlement occurred at 3.55–6.55 m (2–5 m below the subgrade). Fig 14(B) showed that when the installation layer thickness was 0.15 m, the negative value of the settlement occurred at the pavement structure and subgrade. Fig 14(C) showed that when the installation layer thickness was 0.20 m, the time-dependent settlement curve was similar to that of the installation layer thickness of 0.15 m. This phenomenon also indicated that increasing the installation layer thickness reduced settlement and maintained subgrade stability. Hence, the optimization installation layer thickness was 0.15 m, which was beneficial to maintain the settlement stability of the subgrade and economy in manpower and material resources.

**Fig 15:**
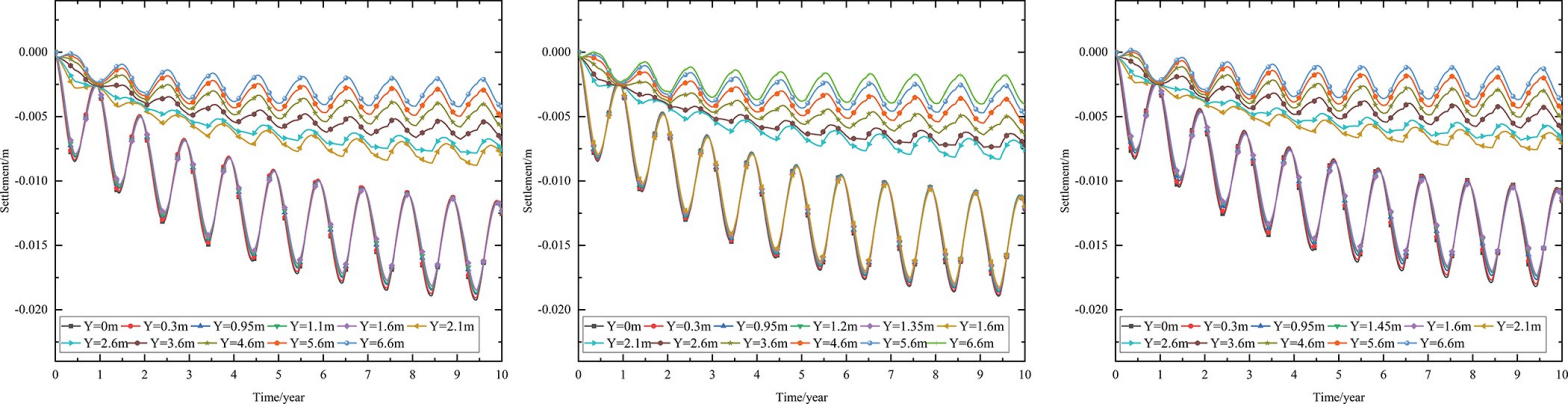
Settlement-time curves of pavement center with the installation layer at (a) the top of the subbase layer, (b) the middle of the subbase layer, and (c) the bottom of the subbase layer.

When the thickness of installation layer is 0.15 m and the 0.50 m-thickness subbase layer is the particle size of 6–8 cm, the time-dependent settlement curves at different location of installation layer are shown in Fig 15.

Fig 15 showed that the negative value of the settlement occurred at the pavement structure and subgrade when the thickness of installation layer was 0.15 m. The maximum settlement of the pavement structure and subgrade decreased when the installation layer varied from the top of the subbase layer to the bottom of the subbase layer. The was because the installation layer prevented the transmission of heat, reduced settlement and maintained subgrade stability. Hence, the optimization location of installation layer was at the bottom of the subbase layer, which was beneficial to maintain the settlement stability of the subgrade.

### 4.3 Changes in settlement under the static load of the aircraft

**Fig 16:**
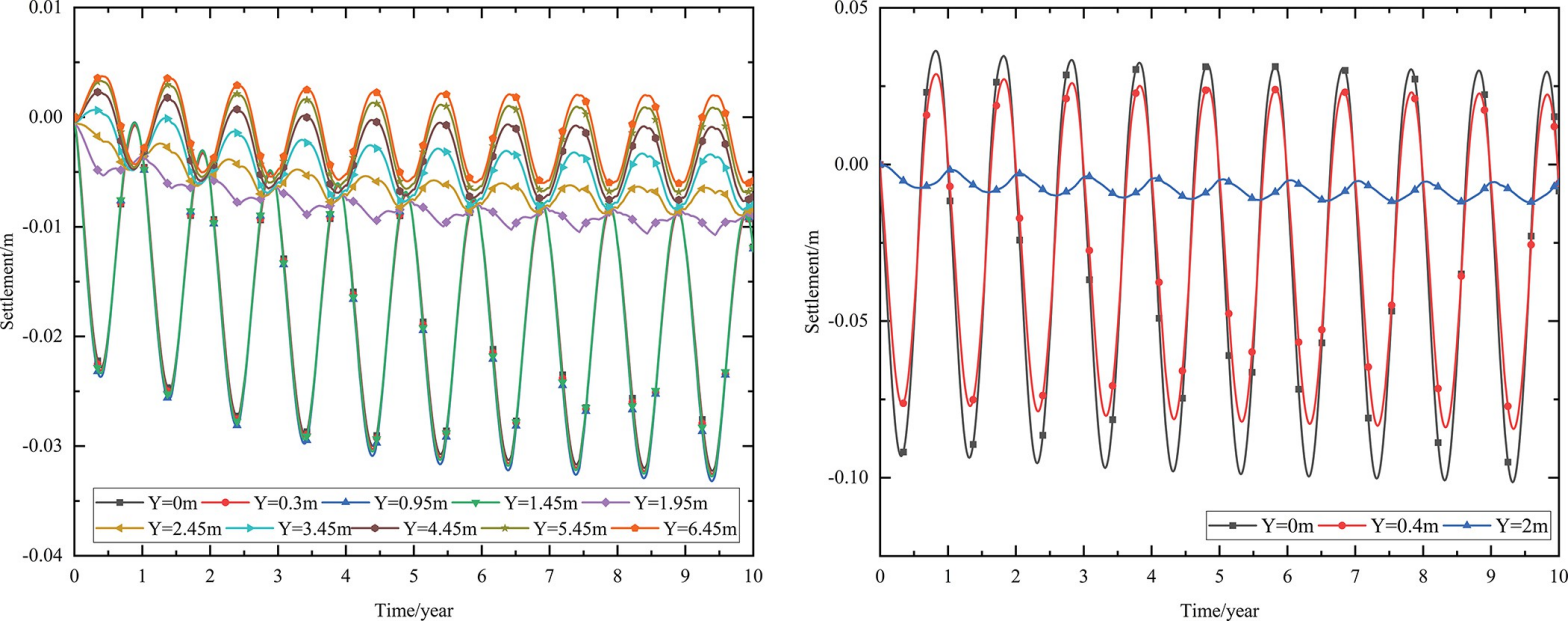
Settlement-time curves under the static load of the aircraft at the (a) pavement center and (b) natural surface center.

**Fig 17:**
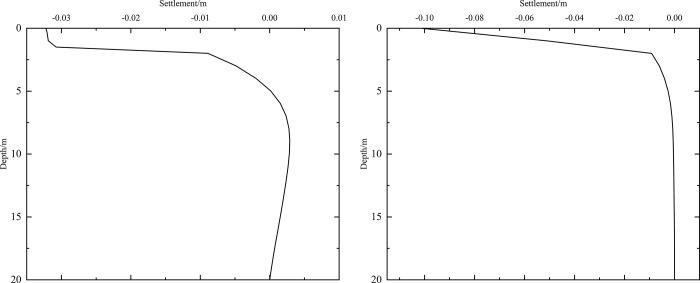
Settlement-depth curves under the static load of the aircraft after 10 years of operation at the (a) pavement center and (b) natural surface center.

As shown in Fig 2, the thickness of the subbase layer remains the same, and the particle size, density, and thermal parameters of the subbase layer are varied, as shown in Table 8. The parameters of the installation layer are listed in Table 9 [34]. When the 0.10 m-thickness installation layer is at the bottom of the subbase layer and the 0.50 m-thickness subbase layer is the particle size of 4–6 cm, the time-dependent curves of settlement are shown in Fig 16 and the depth-dependent curves of settlement are shown in Fig 17.

As shown in Fig 16, as the depth increased, the settlement amplitudes of the pavement and natural surface gradually decreased with phase delay. This phenomenon occurred because pavement settlement required a long time to develop. Moreover, the settlement gradually decreased as depth increased. Finally, the pavement and natural surface reached a new settlement dynamic equilibrium after five or six years. Fig 16(A) and 16(B) indicated that the settlement of the pavement was higher than that of the natural surface at the same depth and time. The difference in the highest settlement of the pavement and natural surface was approximately 0.066 m.

The settlement of the pavement center shown in Fig 16(A) was slightly larger than that shown in Fig 11(A). This was because the settlement caused by the static load of the aircraft was less than that caused by temperature. When the depth was 0.95 m (bottom of the under base layer), the settlement of the pavement varied from -0.034 m to 0 m. When the depth was 1.45 m (bottom of the subbase layer), the settlement of the pavement varied from -0.01 m to 0 m. Therefore, the subbase layer of the graded crushed-rock could significantly reduce the settlement range of the time-dependent curve and was beneficial for maintaining the settlement stability of the subgrade. When the depth was 4.45 m (3 m below the subgrade), the settlement of the pavement had a positive value, indicating that frost deformation occurred. Moreover, frost deformation became increasingly obvious, and the settlement of the positive value increased with increasing depth.

As shown in Fig 17, the ranges of the maximum settlement at the pavement and natural surface decreased as depth increased and tended toward 0 m. Fig 17(A) showed that when the depth ranged from 5.5 m to 20 m, the settlement of the pavement had a positive value of 0–0.004 m. This phenomenon was similar to that shown in Fig 16(A) and also indicated that the subbase layer of the graded crushed-rock was beneficial for reducing the settlement range of the depth-dependent curve to maintain the settlement stability of the subgrade.

When the particle size of the subbase layer was 6–8 cm and 10–15 cm, the evolution laws of the settlement-time and settlement-depth curves were similar to those shown in Figs 16 and 17, respectively. Hence, the data shown in Table 12 were used to further compare the settlement range when the particle size of the subbase layer varied from 4–6 cm to 10–15 cm.

**Table 12:** Settlement of varied subbase layer under the static load of the aircraft.

Parameter	Location	Value		
Particle size (cm)	Pavement center	4–6	6–8	10–15
Thickness (m)	Pavement center	0.5	0.5	0.5
Settlement of 0 m (m)	Pavement center	-0.0323–0	-0.0316–0	-0.0312–0
Settlement of 0.3 m (m)	Pavement center	-0.0326–0	-0.0319–0	-0.0315–0
Settlement of 0.4 m (m)	Natural surface center	-0.0844–0.0289	-0.0844–0.0289	-0.0844–0.0289
Settlement of 0.95 m (m)	Pavement center	-0.0332–0	-0.0325–0	-0.0322–0
Settlement of 1.45 m (m)	Pavement center	-0.0328–0	-0.0321–0	-0.0318–0
Settlement of 1.95 m (m)	Pavement center	-0.0107–0	-0.0103–0	-0.0101–0
Settlement of 2 m (m)	Natural surface center	-0.0120–0	-0.0120–0	-0.0120–0
Settlement of 2.45 m (m)	Pavement center	-0.0090–0	-0.0087–0	-0.0087–0
Settlement of 3.45 m (m)	Pavement center	-0.0084–0.0007	-0.0082–0.0009	-0.0082–0.0009
Settlement of 4.45 m (m)	Pavement center	-0.0077–0.0023	-0.0075–0.0025	-0.0075–0.0025
Settlement of 5.45 m (m)	Pavement center	-0.0069–0.0031	-0.0067–0.0035	-0.0066–0.0035
Settlement of 6.45 m (m)	Pavement center	-0.0061–0.0037	-0.0059–0.0038	-0.0059–0.0039

Table 12 showed when the particle size of the subbase layer was from 4–6 cm to 10–15 cm, the settlement range of the pavement center increased at the depth of 0.3–0.95 m and decreased at the depth of 1.45–6.45 m because the graded crushed-rock maintained the temperature and settlement stability of the subgrade. Moreover, when the depth was the same, the settlement range of the pavement center decreased as the particle size of the graded crushed-rock increased. When the depth exceeded 2.45 m, the settlement of the pavement had a positive value, which might have been caused by frost deformation. The settlement of the negative value in Table 12 was larger than that in Table 11. The settlement of the positive value at a depth of 3.45–6.45 m was slightly smaller than that in Table 11. Thus, the static load of the aircraft increased the negative settlement and reduced the positive settlement caused by frost deformation. However, when the particle size of the subbase layer increased from 6–8 cm to 10–15 cm, the porosity and cooling effect of the subbase layer increased slightly with the growth of the particle size of the subbase layer. And the particle size of 10–15 cm was not suitable for construction and leveling in runway engineering. Therefore, the optimization particle size of the subbase layer was 6–8 cm, which was beneficial to maintain the settlement stability of the subgrade and was convenient for the construction of runway engineering.

**Fig 18:**
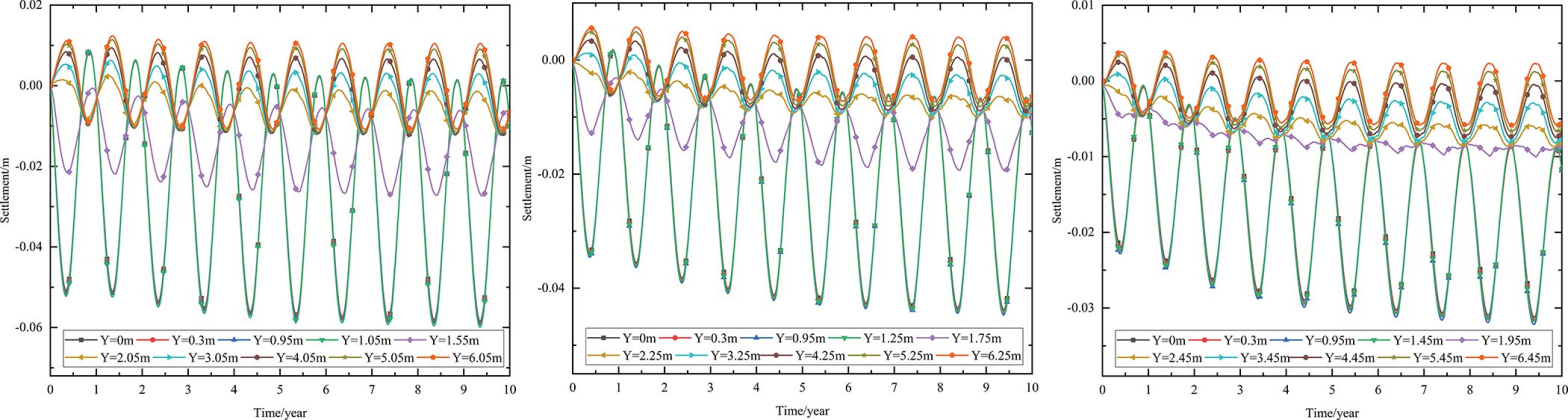
Settlement-time curves of pavement center under the static load of the aircraft at subbase layer thicknesses of (a) 0.10, (b) 0.30, and (c) 0.50 m.

When the 0.10 m-thickness installation layer is at the bottom of the subbase layer and the particle size of the subbase layer is 6–8 cm, the time-dependent settlement curves at different thickness of the subbase layer are shown in Fig 18.

Fig 18 showed that the evolution of settlement under the static load of the aircraft was similar to that of Fig 13. However, the settlement range under the static load of the aircraft was slightly larger than that of Fig 13. The settlement increased over time and then tended to stabilize after 5 to 6 years of operation. When the subbase layer thickness ranged from 0.10 m to 0.50 m, the settlement significantly decreased at the pavement structure layers and slightly reduced at the subgrade. Moreover, when the subbase layer thickness ranged from 0.10 m to 0.30 m, the positive value of the settlement occurred from 3.05 m (2 m below the subgrade) to 4.25 m (3 m below the subgrade). When the subbase layer thickness ranged from 0.30 m to 0.50 m, the settlement had a positive value from 4.25 m (3 m below the subgrade) to 5.45 m (4 m below the subgrade). This phenomenon also indicated that the increase of the subbase layer thickness had an effect on reducing the settlement and maintaining the stability of the subgrade. Hence, the optimization thickness of the subbase layer was 0.5 m, which was beneficial to maintain the settlement stability of the subgrade and ensure the settlement of replacement construction.

**Fig 19:**
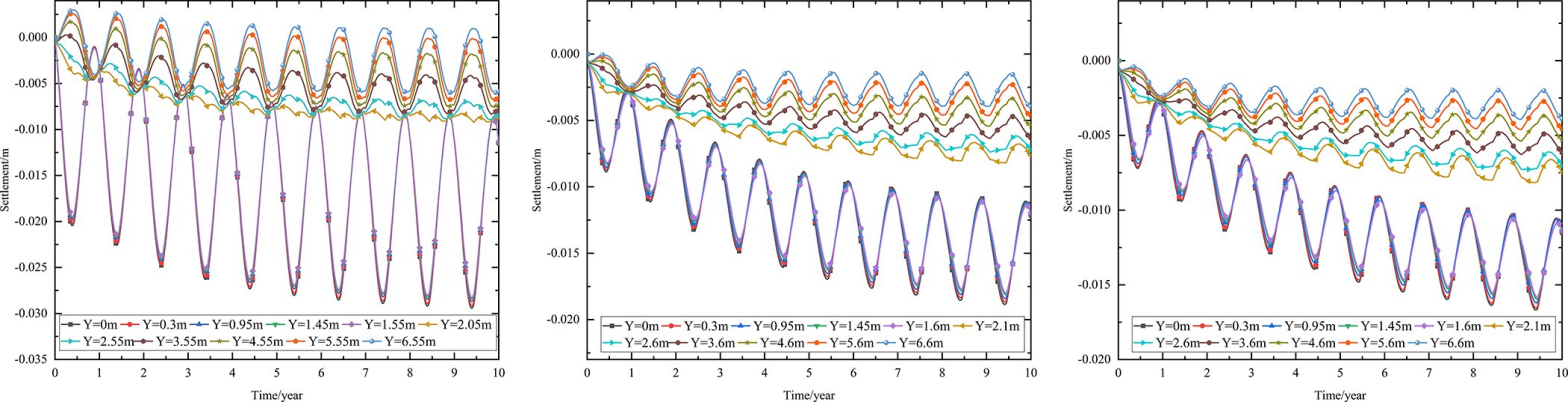
Settlement-time curves of pavement center under the static load of the aircraft at the installation layer thickness of (a) 0.10 m, (b) 0.15 m, and (c) 0.20 m.

When the installation layer is at the bottom of the subbase layer and the 0.50 m-thickness subbase layer is the particle size of 6–8 cm, the time-dependent settlement curves at different thickness of installation layer are shown in Fig 19.

Fig 19 represented that the settlement decreased as installation layer thickness increased and the absolute value of the settlement was slightly larger than that of Fig 14. Because the increase of the installation layer thickness was beneficial to prevent the transmission of heat and reduce pavement settlement. Moreover, the settlement of Fig 19 caused by the temperature and the static load of the aircraft.

Fig 19(A) showed that when the installation layer thickness was 0.10 m, the positive value of the settlement occurred at 4.55–6.55 m (3–5 m below the subgrade). Fig 19(B) showed that when the installation layer thickness was 0.15 m, the negative value of the settlement occurred at the whole runway structure. Fig 19(C) showed that when the installation layer thickness was 0.20 m, the time-dependent settlement curve was similar to that of the installation layer thickness of 0.15 m. This phenomenon indicated that increasing the installation layer thickness reduced settlement and maintained subgrade stability. Hence, the optimization installation layer thickness was 0.15 m, which was beneficial to maintain the settlement stability of the subgrade and economy in manpower and material resources.

**Fig 20:**
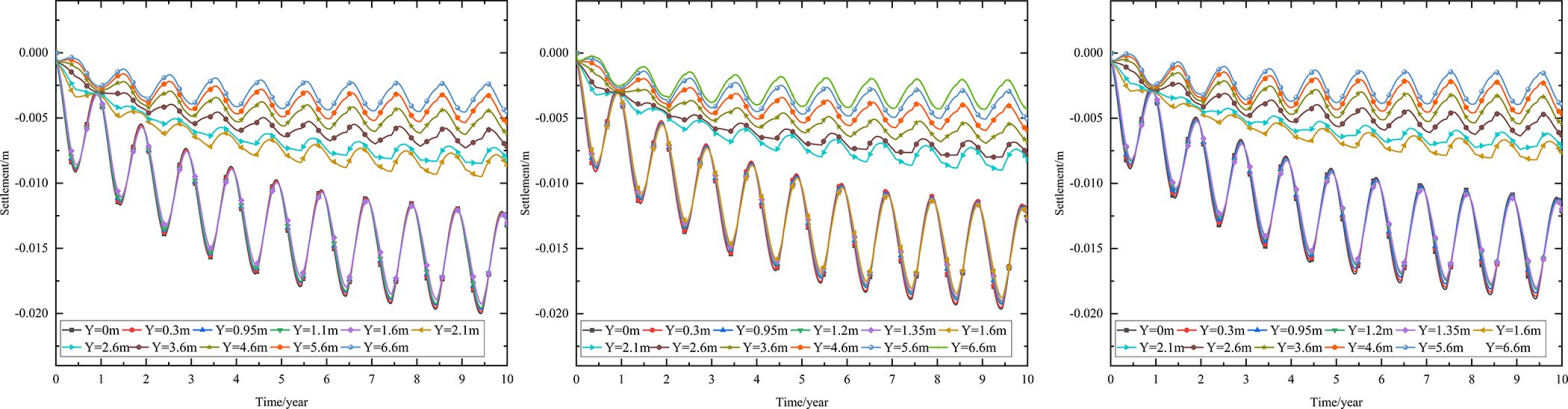
Settlement-time curves of pavement center under the static load of the aircraft with the installation layer at (a) the top of the subbase layer, (b) the middle of the subbase layer, and (c) the bottom of the subbase layer.

When the thickness of installation layer is 0.15 m and the 0.50 m-thickness subbase layer is the particle size of 6–8 cm, the time-dependent settlement curves at different location of installation layer are shown in Fig 20.

Fig 20 showed that the negative value of the settlement occurred at the whole runway structure and the absolute value of the settlement was slightly larger than that of Fig 15. Because the settlement of Fig 20 caused by the temperature and the static load of the aircraft. The maximum settlement of the pavement structure and subgrade decreased when the installation layer varied from the top of the subbase layer to the bottom of the subbase layer. The was because the installation layer prevented the transmission of heat, reduced settlement and maintained subgrade stability. Hence, the optimization location of installation layer was at the bottom of the subbase layer, which was beneficial to maintain the settlement stability of the subgrade.

## 5. Conclusions

In this study, a numerical model of the structure of the pavement and subgrade in permafrost regions is developed through a comparison with previous models in terms of the characteristics of the temperature field, frozen layer depth, and settlement without considering aircraft load. This study also analyzes the temperature and settlement of different pavement structures. The main conclusions are as follows:

1. When the combination of graded crushed-rock layer and insulation layer is included in the subgrade, the temperature amplitudes of the pavement and natural surface gradually decrease with some phase delay as depth increases. The temperature amplitudes of the pavement at the bottom of the combination of graded crushed-rock layer and insulation layer vary significantly and are smaller than those at the bottom of the base layer. The temperature ranges of the pavement and natural surface decrease with increasing depth and tend to be constant. The cooling effect of the combination of graded crushed-rock layer and insulation layer is enhanced with an increase in the particle diameter and thickness of graded crushed-rock layer and the thickness of insulation layer. Moreover, the location of installation layer has significant influence on the temperature of the subbase layer, but has little influence on the temperature of the subgrade.
2. The combination of graded crushed-rock layer and insulation layer can significantly reduce the settlement range of the time-dependent curve and maintain the settlement stability of the subgrade. The settlement range of the pavement center decreases as the particle diameter and thickness of graded crushed-rock layer and the thickness of insulation layer increases. When the depth exceeds 2.45 m, pavement settlement is a positive value due to the effect of frost deformation. The maximum settlement of the pavement structure and subgrade decreases when the installation layer varies from the top of the subbase layer to the bottom of the subbase layer.
3. The evolution of the settlement under the static load of the aircraft is similar to that of the no-aircraft case. The settlements at the pavement and natural surface centers decrease with increasing depth and tend to zero. The settlement decreases with increasing the particle diameter and thickness of graded crushed-rock layer and the thickness of insulation layer. The maximum settlement of the pavement structure and subgrade decreases when the installation layer varies from the top of the subbase layer to the bottom of the subbase layer. However, the graded crushed-rock layer particle size of 10–15 cm is not suitable for construction and leveling in runway engineering and increasing the porosity and cooling effect of the subbase layer slightly. And the time-dependent temperature and settlement curves are similar at the installation layer thickness of 0.15 m and 0.20 m. The optimization particle size of the subbase layer is 6–8 cm, the optimization thickness of the subbase layer is 0.5 m, the optimization thickness of the installation layer is 0.15 m, and the optimization location of installation layer is at the bottom of the subbase layer.
